# Compensatory shifts in visual perception are associated with hallucinations in Lewy body disorders

**DOI:** 10.1186/s41235-017-0063-6

**Published:** 2017-05-24

**Authors:** Alan Robert Bowman, Vicki Bruce, Christopher J. Colbourn, Daniel Collerton

**Affiliations:** 1 Department of Clinical Psychology, School of Health and Social Care, Teesside University, Middlesbrough, TS1 3BX UK; 2 Newcastle University, School of Psychology, Ridley Building 1, Queen Victoria Road, Newcastle-Upon-Tyne, NE1 7RU UK; 3 Northumberland, Tyne, & Wear NHS Foundation Trust, Clinical Psychology Department, Bensham Hospital, Gateshead, UK

**Keywords:** Visual hallucinations, Signal detection theory, Lewy body disorders, Parkinson’s disease, Dementia, Pareidolia

## Abstract

Visual hallucinations are a common, distressing, and disabling symptom of Lewy body and other diseases. Current models suggest that interactions in internal cognitive processes generate hallucinations. However, these neglect external factors. Pareidolic illusions are an experimental analogue of hallucinations. They are easily induced in Lewy body disease, have similar content to spontaneous hallucinations, and respond to cholinesterase inhibitors in the same way. We used a primed pareidolia task with hallucinating participants with Lewy body disorders (n = 16), non-hallucinating participants with Lewy body disorders (n = 19), and healthy controls (n = 20). Participants were presented with visual “noise” that sometimes contained degraded visual objects and were required to indicate what they saw. Some perceptions were cued in advance by a visual prime. Results showed that hallucinating participants were impaired in discerning visual signals from noise, with a relaxed criterion threshold for perception compared to both other groups. After the presentation of a visual prime, the criterion was comparable to the other groups. The results suggest that participants with hallucinations compensate for perceptual deficits by relaxing perceptual criteria, at a cost of seeing things that are not there, and that visual cues regularize perception. This latter finding may provide a mechanism for understanding the interaction between environments and hallucinations.

**Electronic supplementary material:**

The online version of this article (doi:10.1186/s41235-017-0063-6) contains supplementary material, which is available to authorized users.

## Significance

Visual hallucinations are a common symptom of Lewy body disorders (LBD) and are associated with increased distress in both patients and carers. There is a need to develop an understanding of the mechanisms underpinning visual hallucinations to inform the management and treatment of this symptom. We report the first investigation into the effects of visual priming on a complex visual illusion that is posited to be a useful analogue of visual hallucinations. We show that visual hallucinations are associated with a tendency to accept illusory perceptions as real, in order to avoid missing true perceptions. This finding suggests that the underlying mechanism of visual hallucinations in Lewy body disorders may be one of compensation, rather than deficit. Furthermore, the results suggest that environmental cues can influence a person’s tendency to perceive visual information as real. The findings support a novel mechanism for explaining the interaction between environment and hallucinatory episodes in these disorders. An implication for interactive models of visual perception is that in the face of perceptual (bottom-up) impairment, top-down factors act to compensate for this impairment. By extension, providing patients with LBD with rich visual environments that eliminate visual ambiguity, and providing environmental cues to aid their perception, may reduce hallucination frequency in this population.

## Introduction

Visual hallucinations—the involuntary experience of seeing something that is not veridically present while awake (Collerton, Perry, & McKeith, 2005)—are a common occurrence in Lewy body disorders (LBD) such as Parkinson’s disease, Parkinson’s disease dementia, and dementia with Lewy bodies (Fénelon & Alves, 2010; Ferman et al., 2013; Kitayama, Wada-Isoe, Nakaso, Irizawa, & Nakashima, 2007) as well as a number of other disorders and normal states. The psychosocial impact of visual hallucinations can be high in LBD and has been associated with increased distress (Mosimann et al., 2006), co-morbid mental health problems (Bjoerke-Betheussen, Ehrt, Rongve, Ballard, & Aarsland, 2012), and increased carer distress (Lee, McKeith, Mosimann, Ghosh-Noydal, & Thomas, 2013).

Current theoretical explanations of visual hallucinations, while differing in emphasis, broadly agree that impairments in both visual perception and attention are implicated (Collerton & Mosimann, 2010). Models such as the attentional network (Shine, Halliday, Naismith, & Lewis, 2011), Attention, Input, Modulation (Diederich, Goetz, & Stebbins, 2005), and Perception Attention Deficit (Collerton et al., 2005) all propose to differing degrees that combined impairments in both “bottom-up” perceptual functions and “top-down” attentional processes lead to the intrusion of erroneous object representations—hallucinations—into the visual scene.

Consistent with these models, combined impairments in top-down and bottom-up processes are risk factors for visual hallucinations in LBD (Barnes & Boubert, 2008; Bronnick, Emr, Tekin, Haughen, & Aarsland, 2011; Gallagher, Parkkinen, O’Sullivan, Spratt, Shah et al., 2011; Imamura, Wada-Isoe, Kitayama, & Nakashima, 2008; Meppelink, De Jong, Teune, & Van Laar, 2009; Ozer, Meral, Hanoglu, Ozturk, Cetin et al., 2007; Ramírez-Ruiz, Junqué, Martí, Valldeoriola, & Tolosa, 2006, 2007; Straughan, Collerton, & Bruce, 2016). This focus on factors within participants, however, neglects potential interactions with the environment and does not provide an explanation of why hallucinatory episodes tend to occur at specific times and locations and to be tightly integrated with the visual environment (Collerton, Taylor, Tsuda, Fujii, Nara et al., 2016).

**Fig. 1:**
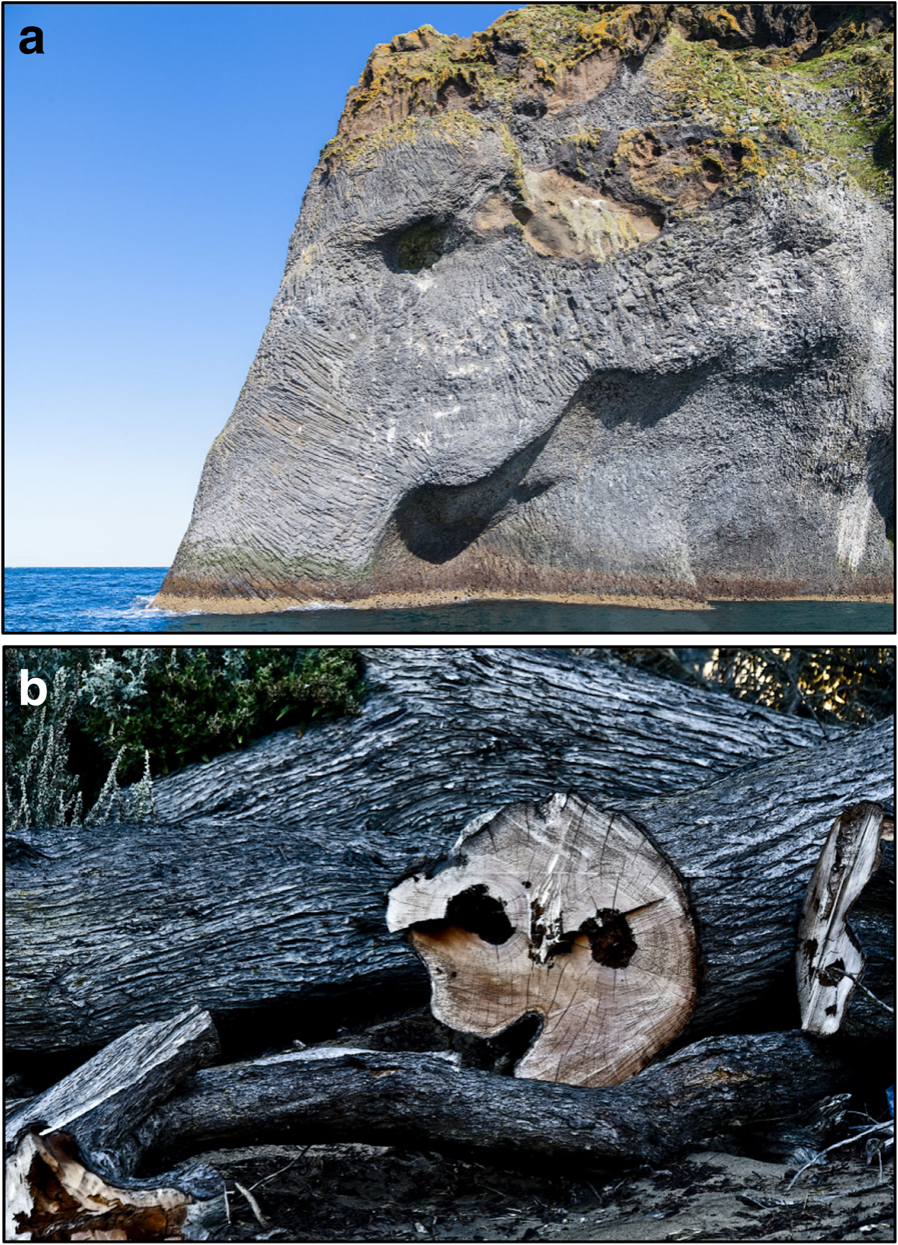
Examples of naturally occurring pareidolia: (**a**) elephant rock (Delso, 2014); (**b**) face in tree trunk (Denyer, n.d.)

Investigating individual hallucinations, given their unpredictable episodic nature, is difficult. The recent development of the pareidolia task as an experimental analogue of visual hallucinations has addressed this gap (Uchiyama, Nishio, Yokoi, Kirayama, Imamura et al., 2012). Pareidolia are a class of visual illusion whereby a visual object is perceived in visual information which is itself meaningless, ambiguous, or bears no veridical relationship to what is perceived by the viewer. “Seeing faces in clouds” is a naturally occurring example (see Fig. 1 for examples of naturally occurring pareidolia). Studies have demonstrated considerable overlap in the phenomenology of pareidolia and visual hallucinations (Uchiyama, Nishio, Yokoi, Hosokai, Takeda et al., 2015). Moreover, hallucination severity is positively correlated with experimental pareidolia frequency and both respond to cholinesterase inhibitors (Yokoi, Nishio, Uchiyama, Shimomura, Iizuka et al., 2014). Uchiyama et al. (2012) found that the number of illusory perceptions in pareidolia images was able to discriminate between participants with dementia with Lewy bodies and Alzheimer’s disease with 100% sensitivity and a specificity of 88%.

Signal detection theory offers a potentially fruitful avenue for testing interactional models, because it provides a way to measure bottom-up perceptual processes (i.e., sensitivity) and higher order “top-down” processes (i.e., response bias) at the same time (Dolgov & McBeath, 2005). Yokoi et al. (2014) applied signal detection theory to their pareidolia test by varying the absence/presence of real visual objects among ambiguous visual noise. They demonstrated that, compared with participants with Alzheimer’s disease, participants with dementia with Lewy bodies had significantly reduced ability to discriminate visual “signal” from “noise,” and were more prone to report the presence of a visual object when one was not present. These perceptual effects were suggested to be related to the risk of visual hallucinations.

A recent study from our group (Straughan et al., 2016) examined the role of priming of visually ambiguous images (e.g., silhouettes, fragmented images) in hallucinating and non-hallucinating participants with Parkinson’s disease (this was a separate sample from the current study). Significant priming effects emerged across both groups, demonstrating the preserved priming in participants with LBD. Hallucinating participants were significantly more impaired in resolving visually ambiguous images. Because all trials in this study involved the presence of an object (i.e., there was always something to “see”), the question remains as to what happens when hallucinating participants are primed in the absence of a veridical percept. Pareidolia offer a way to investigate this, as the absence/presence of an object within visual noise can be manipulated.

The current study aimed to extend the findings of Straughan et al. (2016) and Yokoi et al. (2014) by assessing differences between patients with and without hallucinations and by adding associative priming to experimentally examine the effects of environmental factors.

## Materials and methods

### Experimental design

A cross-sectional design was used to investigate the effects of priming in an experimental pareidolia task in three groups: healthy older adults (HC), people with LBD who do not experience visual hallucinations (VH-), and people with LBD who experience recurrent complex visual hallucinations (VH+). Allocation to group was non-random and made on the basis of diagnosis (presence/absence of LBD) and presence/absence of visual hallucinations.

An a priori power calculation was conducted using G*Power software (Faul, Erdfelder, Lang, & Buchner, 2007). This indicated a required sample size of n = 60 (20 per group) to detect a large effect size (*f* = 0.4), with power = 0.95, α = 0.05.

### Participants

Ethical approval for the study was obtained from the National Health Service North East Research Ethics Committee (committee reference no: 14/NE/1104, project ID: 144270). All participants gave their informed consent to participate, in accordance with the Declaration of Helsinki.

Participants with LBD (Parkinson’s disease, Parkinson’s disease with dementia, or dementia with Lewy bodies) were recruited from movement disorder clinics and from local support groups run by the charity Parkinson’s UK. Participants experiencing visual hallucinations within the past month as assessed by the North East Visual Hallucinations Inventory (NEVHI; Mosimann, Collerton, Dudley, Meyer, Graham et al., 2008) formed the VH+ group, whereas participants not experiencing VH within the past month formed the VH– group. This “one month rule” was applied to differentiate actively hallucinating participants from those who may have had hallucinations in the past, but who no longer experience them due to effective treatment of this symptom. Friends and relatives of people with LBD formed the HC group.

Participants were not eligible for the study if they had any psychiatric or other medical condition associated with visual hallucinations. Referrers to the study were advised that participants required a best corrected visual acuity of > 0.4 (acuity in decimalized Snellen form, as measured by the Landolt-C optotype). Participants in the VH+ and VH– groups were eligible to take part in the study if they had a confirmed diagnosis (made by a neurologist) of Parkinson’s disease, Parkinson’s disease dementia, or dementia with Lewy bodies, and were stable on all medications (for Parkinsonism or otherwise) for at least three months. Participants who scored below 20 on the Montreal Cognitive Assessment (MoCA; Nasreddine, Phillips, Bédirian, Charbonneau, Whitehead et al., 2005) were excluded from the study a priori (n = 3) in order to exclude participants who were unable to give informed consent or who might struggle to complete the experimental tasks. The characteristics of the final groups are shown in Table 1.

**Table 1:** Sample characteristics

	HC	VH–	VH+
n	20	19	16
Age (years)	69.3 (6.55)	66.5 (8.02)	71.06 (6.75)
Females (n)^a^	14	7	3
Visual acuity^a^	0.93 (0.33)	0.97 (0.42)	0.66 (0.22)
Cognitive ability^b^	27.9 (1.66)	25.6 (2.89)	22.7 (2.47)
Medications (n)	–	4.84 (3.59)	6.25 (3.26)
Parkinsonism severity^c^	–	33.4 (18.47)	53.8 (18.58)

^a^ *p* < 0.05

^b^ *p* < 0.001

^c^ *p* < 0.01

### Measures and analysis

#### Primary measures

**Fig. 2:**
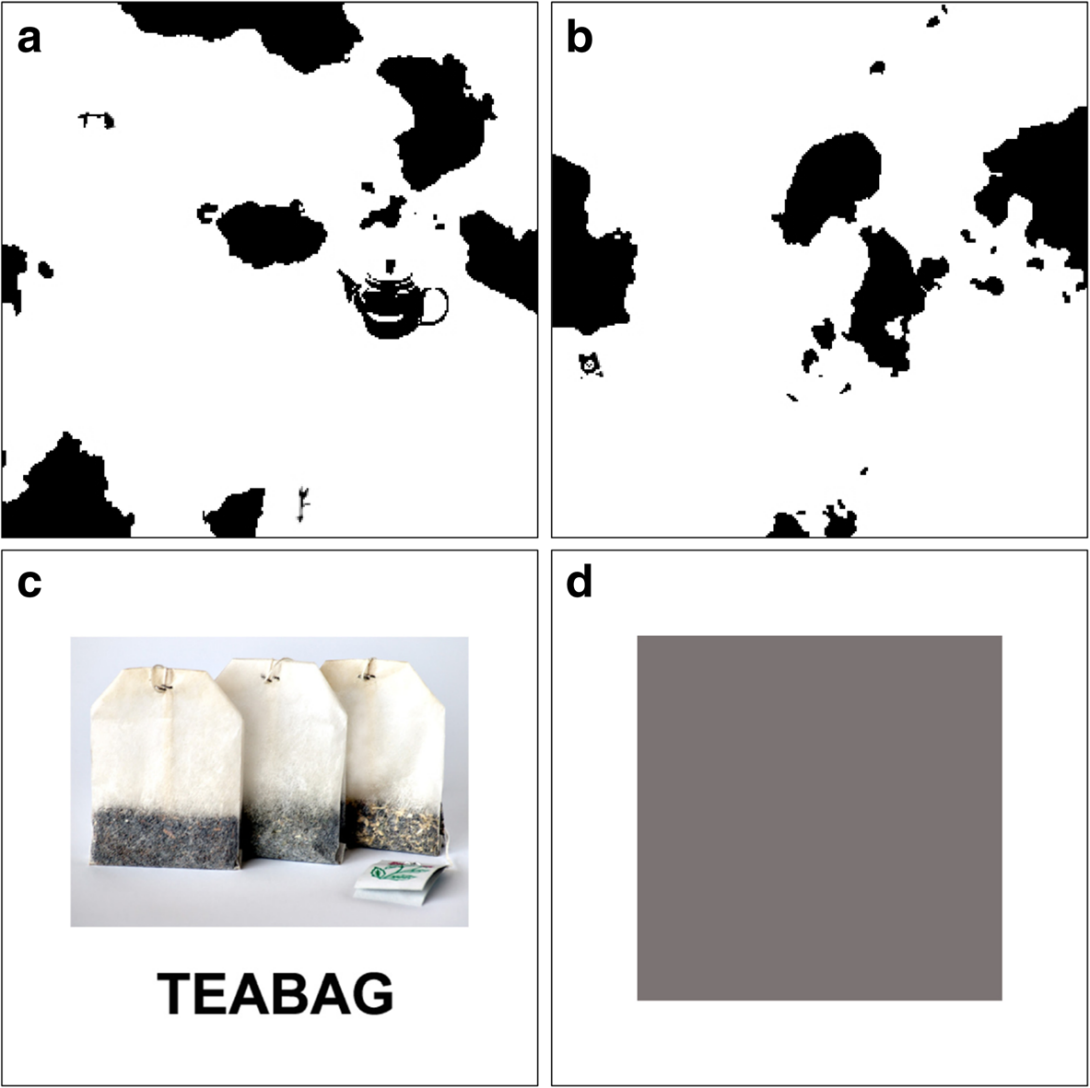
Exemplar stimuli from the pareidolia experiment. Stimuli include a signal trial (target item is a teapot, mid-right of visual scene) (**a**), a noise trial (**b**), a prime item (**c**), and a “no prime” item (**d**)

The two primary signal detection variables of interest were sensitivity (the ability to discriminate visual signals from visual noise) and response bias (the tendency to report the presence of a signal, regardless of whether one is present). These variables were measured using a novel pareidolia test involving the presentation of visually ambiguous images (Fig. 2). The experiment consisted of 40 trials. In half of the trials, the pareidolia stimulus featured a picture of an object that had been visually degraded to make it ambiguous (“signal trials;” Fig. 2a). The remaining half trials featured “blank” pareidolia stimuli with no embedded visual object in them (“noise trials;” Fig. 2b). In order to investigate the role of priming, half of the trials involved presenting a semantically related prime prior to the pareidolia stimulus (“primed trials;” Fig. 2c), while the remaining half featured were not primed (“unprimed trials;” Fig. 2d). This gave rise to four types of trial: (1) primed, signal trial; (2) unprimed, signal trial; (3) primed, noise trial; and (4) unprimed, noise trial. Both the order of trials and the prime type (primed, unprimed) were counterbalanced.

In accordance with signal detection theory two measures were calculated for the analysis; perceptual sensitivity (*d’*) and response bias (*c*). The former was calculated using the formula *d’ = z*(hit rate) + *z*(false alarm rate)/2 (Macmillan & Creelman, 2005). Hit rate is defined by number of correct responses (i.e., correctly reporting the presence of an object that was veridically present in a trial) divided by the number of trials. False alarm rate is defined as the number of illusory responses (i.e., perceiving a visual object during a trial where no objects were present) divided by the number of trials.

Higher *d’* scores are indicative of a participant being more able to separate visual signals from visual noise. Criterion measures the likelihood of reporting the presence of a signal regardless of what is veridically present, and was calculated using the formula *c* = -(*z*(hit rate) + *z*(false alarm rate))/2 (Macmillan & Creelman, 2005). A *c* of < 0 indicates a more “liberal” response bias (i.e., participants are more prone to “hits” at the cost of also making more “false alarms”). A *c* of > 0 indicates a more “conservative” response bias; participants are less likely to make “false alarms,” at the expense of also missing veridically present visual objects.

For the purposes of signal detection analysis, trials are scored in a binary manner (i.e., the participant either has a false alarm or does not). It is possible that hallucinating participants may perceive multiple illusory perceptions within the same trial—something that signal detection analysis alone is unable to account for. Because of this, the absolute number of illusory perceptions per trial (pareidolia rate) was also recorded.

#### Descriptive measures

Overall cognitive ability was measured using the Montreal Cognitive Assessment (Nasreddine et al., 2005). Presence and phenomenology of visual hallucinations was determined using the NEVHI (Mosimann et al., 2008), a semi-structured interview designed to assess the presence, phenomenology, frequency, duration, and emotional impact of visual hallucinations. The Landolt-C optotype from the Freiburg Visual Acuity Test (Bach, 2014) was administered to assess best corrected decimal visual acuity at 200 cm. VH– and VH+ participants completed the motor examination subsection of the Unified Parkinson’s Disease Rating Scale (UPDRS; Fahn & Elton, 1987) to establish the severity of Parkinson’s symptoms. The motor examination was composed of 13 test items that assess rigidity, tremor, posture, manual dexterity, and facial expression.

#### Procedure

Testing was conducted in participants’ homes on a 13” Apple © Macbook Pro running at a resolution of 1280 × 800 pixels. Participants were positioned about 50 cm from the screen in a room with good illumination. They were told that they would be presented with ambiguous images that would sometimes include pictures of visual objects and were asked to report whether they detected a visual object and, if so, report what it was they saw. Responses were recorded manually by the experimenter. Reaction times were not measured due to the difficulties of accurately recording this variable in participants with movement disorders.

When a participant perceived a visual object, they were asked to point to its location on the screen or, alternatively (if their movement difficulties precluded this), state which quadrant of the screen they saw the object. This step was taken to account for the fact that participants may perceive an illusory object in addition to or instead of the object that is present in the trial. For example, in a signal trial featuring a picture of a teapot, a response would be coded as correct if the participant reported seeing a teapot and indicated its presence in the correct spatial location on the screen. If on the other hand, the participant perceived a teapot in a different spatial location, the response would be coded as a false alarm. If a participant experienced both a veridical and an illusory perception within the same trial, the trial would be recorded as a correct response for the purposes of signal detection analysis, but the number of illusory responses in total was also recorded in order to calculate pareidolia rate.

#### Statistical analysis

Data analysis was conducted using SPSS version 19. Preliminary analyses of the data were conducted to establish whether parametric assumptions were met (i.e., normality, homogeneity of variance). Where they were, parametric tests (ANOVA, *t*-tests) were carried out. In the event that parametric assumptions were violated, non-parametric alternatives were applied (Mann–Whitney U, Kruskal–Wallis, Wilcoxon). All statistical tests were two-tailed, with an alpha level of *p* < 0.05 to determine statistical significance.

## Results

### Sample characteristics

Key sample characteristics are summarized in Table 1. The total sample size was 55, approaching the target of 60 for adequate statistical power. The three participant groups were matched in terms of age and education level. The two clinical groups were also matched in terms of number of medications. Hallucinating participants exhibited poorer visual acuity, lower cognitive ability, and increased disease severity, relative to the other groups. Two participants in the VH– group reported historical visual hallucinations prior to diagnosis and treatment for their condition. These two participants were included in the VH– group because they reported being stable on their medication and hallucination-free for over one year.

Two hallucinating participants and two non-hallucinating participants exhibited visual acuity scores below the 0.4 cutoff. These participants were included in the final analysis to preserve sample size and because there was an equal number of these participants in hallucinating and non-hallucinating groups. Additional analyses of the influence of visual acuity was conducted (see “Visual acuity” section) to examine the possible influence of this variable in more detail. Non-hallucinating LBD participants and healthy controls were comparable on all demographic variables with the exception of scores on the Montreal Cognitive Assessment, which was significantly higher in the healthy controls.

Diagnoses of the non-hallucinating LBD patients consisted of Parkinson’s disease (n = 17), Parkinson’s disease dementia (n = 1), and probable dementia with Lewy bodies (n = 1). The hallucinating LBD participants were composed of Parkinson’s disease (n = 5), Parkinson’s disease dementia (n = 8), probable dementia with Lewy bodies (n = 2), and possible dementia with Lewy bodies (n = 1).

### Phenomenology

#### Hallucinations

All participants in the VH+ group reported recurrent complex visual hallucinations within the last four weeks of testing. In addition, four also reported simple visual hallucinations such as spots, flashes, or geometric shapes. No members of the non-hallucination groups reported complex visual hallucinations. Of the HC participants, two reported simple visual hallucinations in the form of spots (“visual floaters”) from previous cataract surgery. Three VH– participants reported simple visual hallucinations (spots, flashes of light, shadows, geometric shapes).

For most hallucinating participants, their hallucinations started several years ago (75%) and had a duration lasting between 1 min and 1 h (69%). Hallucinations were for the most part realistically colored (63%), sized (81%), and had realistic motion (56%). Over half of the participants (56%) did not find their hallucinations distressing or frightening, although many did find them unpleasant/irritating (63%).

Consistent with the typology of visual hallucinations in LBD, hallucination content consisted primarily of people, animals, and objects (87.5%). Two hallucinating participants reported seeing body parts (e.g., hand, head) and three participants reported hallucinations classified as “other” (e.g., objects that appear half human, half inanimate).

#### Pareidolia

As Table 3 indicates, hallucinating participants perceived a significantly higher number of pareidolia than did non-hallucinating participants. Consistent with previous findings (Uchiyama et al., 2012), pareidolia of people and/or animals accounted for a large proportion of illusory responses in the hallucinating group (93.8% of hallucinating participants reported seeing people, animals, and/or objects). Common examples included, seeing men, women, dogs, and cats. Body parts (e.g., hand. head) were perceived by 68.8% of hallucinating participants and inanimate objects (e.g., hat, comb) by 87.5% of this group. Pareidolia content was more varied in the non-hallucinating groups, with 38.5% of participants perceiving people and/or animals. Body parts were perceived by 32.1% of this group and inanimate objects by 43.6% of the group. Note that these categories are not mutually exclusive and it was common for hallucinating participants in particular to report multiple pareidolia of different types within the same trial (e.g., cat and a face). Twenty-five percent of hallucinating participants and 5.1% of non-hallucinating participants reported responses classified as “other.” For example, island formations or symbols.

### Sensitivity

Hit rate, false alarm rate, sensitivity, and response bias are summarized in Table 2. The VH+ group had a considerably lower sensitivity index than either of the non-hallucinating groups. Mann–Whitney U tests revealed that there was a significant difference in sensitivity between the VH+ and HC group during both primed (*U* = 3.5, *p* < 0.001) and unprimed (*U* = 27.0 *p* < 0.001) trials. Similarly, significant differences in sensitivity emerged between the VH+ and VH– group during primed (*U* = 2.5, *p* < 0.001) and unprimed (*U* = 26.0, *p* < 0.001) trials. No significant differences emerged between the two non-hallucinating groups.

**Table 2:** Hit rate, false alarm (FA) rate, sensitivity (d'), and response bias (c) across groups

Group	Unprimed	Primed	Unprimed	Primed
	Hit rate		FA rate	
HC	0.75 (0.20)	0.88 (0.14)^a^	0.14 (0.15)	0.05 (0.10)^b^
VH-	0.72 (0.19)	0.83 (0.13)^b^	0.08 (0.13)	0.07 (0.07)
VH+	0.53 (0.24)^c^	0.59 (0.26)^c^	0.63 (0.38)^c^	0.49 (0.35)^c^
	D prime		Criterion	
HC	2.37 (0.8)	2.85 (0.54)^b^	−0.04 (0.41)	0.02 (0.2)
VH-	2.33 (0.84)	2.68 (0.39)	0.11 (0.31)	0.03 (0.26)
VH+	0.46 (1.15)^c^	0.64 (0.91)^c^	0.28 (0.55)^d^	−0.14 (0.68)

^a^Significant effect of prime (*p* < 0.05)

^b^Significant effect of prime (*p* > 0.05)

^c^VH+ significantly different to HC and VH– (*p* < 0.001)

^d^VH+ significantly different to VH– group only (*p* < 0.001)

A Wilcoxon signed ranks test indicated that the addition of a prime significantly improved sensitivity in the HC group (*z* = –2.39, *p* = 0.02). The VH– and VH+ groups demonstrated increased sensitivity in the presence of a prime, but this was not statistically reliable.

### Visual acuity

As Table 1 indicates, hallucinating participants had poorer visual acuity than their non-hallucinating counterparts. In addition, four participants were included in the analysis with decimal visual acuity below 0.4. It is possible that the observed differences in sensitivity between hallucinating and non-hallucinating participants is simply a reflection of differences in visual acuity. To rule this out, the non-hallucinating groups were aggregated and divided into “low acuity” (n = 20) and “high acuity” (n = 19) groups, by means of a median split (median acuity of non-hallucinators = 0.92). The visual acuity of the low acuity, non-hallucinating participants (0.64, *SD* = 0.17) was comparable to that of hallucinating participants (0.66, SD = 0.22, U = 152.5, *p* = 0.81). In contrast, significant differences in sensitivity (d’) emerged between these groups. For unprimed trials, hallucinating participants exhibited significantly lower sensitivity scores (0.46, *SD* = 1.15), than low acuity, non-hallucinators (2.19, *SD* = 0.93, *U* = 35.5, *p* < 0.001). This reduced level of sensitivity in hallucinating participants was also found for primed trials (0.64, *SD* = 0.91, versus 2.63, *SD* = 0.49, *U* = 5.00, *p* < 0.001)

### Response bias

Within-subject comparisons of unprimed versus primed trials revealed no significant effect for any group. Between-group comparisons of response bias revealed that during unprimed trials, hallucinating LBD patients exhibited a reliably lower (more liberal) response bias than non-hallucinating LBD patients (*U* = 84.0, *p* = 0.02), but not healthy controls. The introduction of a prime increased the response bias of the VH+ group to the levels of the HC and VH– groups (i.e., the significant difference between VH+ and VH– was eliminated).

### Pareidolia rate

Absolute number of false alarms per trial (pareidolia rate) was also explored, in order to account for participants who perceived multiple illusory objects within the same trial. While priming reduced pareidolia rate in hallucinating participants (Table 3), the rates of pareidolia in this group remained significantly higher than non-hallucinating participants (*p* < 0.001 across all trial types).

**Table 3:** Within-group and between-group comparisons of pareidolia rate

Group	Unprimed	Primed
	Pareidolia rate (noise trials)	
HC	1.35 (1.63)	0.55 (0.89), *p* = 0.05
VH–	0.84 (1.38)	0.53 (0.70), *p* = 0.16
VH+	6.13 (3.54)	4.80 (3.17), *p* = 0.08
	Pareidolia rate (signal trials)	
HC	0.00 (0.00)	0.10 (0.31) *p* = 0.22
VH–	0.05 (0.23)	0.05 (0.23), *p* = 1.00
VH+	3.50 (2.92)	2.38 (2.33), *p* = 0.02

## Discussion

The results corroborate previous findings that pareidolia are a good analogue to visual hallucinations (Uchiyama et al., 2015; Yokoi et al., 2014). In the present study, there was considerable overlap between the phenomenology of both and hallucinating participants were more prone to pareidolia than their non-hallucinating counterparts. Thus, the previously established difference between groups prone to hallucinations (LBD) and those not (Alzheimer’s disease or controls) is specific to those participants who do hallucinate.

Non-hallucinating LBD participants were indistinguishable from healthy controls on both signal detection measures. Consistent with the hypothesis that hallucinations are associated with an impaired ability to separate visual signal from noise, hallucinating LBD participants displayed significantly lower perceptual sensitivity than the non-hallucinating groups.

Hallucinating LBD participants exhibited a significantly lower response criterion during unprimed trials, in comparison to non-hallucinating LBD participants. The presentation of a prime item eliminated this difference. On the other hand, no significant priming effects emerged when unprimed versus primed trials were compared within groups.

During the experiment, over half (9/16) hallucinating participants reported multiple pareidolia within a single trial. Traditional signal detection measures are unable to account for this fully, as they measure absence/presence of illusory perception, rather than absolute number of perceptions within a trial. Using signal detection analysis may therefore be overly conservative in this context. When the absolute number of illusory perceptions (pareidolia rate) was considered, it emerged that the presentation of a prime item reduced the number of pareidolia experienced by hallucinating participants, although relative number of pareidolia in this group remained high in comparison to non-hallucinators.

The lower criterion score in hallucinating participants during unprimed trials may be indicative of a downward shift in response bias in order to compensate for poor perceptual sensitivity. In other words, hallucinating LBD participants were more “liberal” in their response bias to avoid missing veridical percepts, at the cost of occasionally accepting visual noise as a percept. This is consistent with the proposal of Dolgov and McBeath (2005) that hallucinations are underpinned by a more relaxed response bias in order to maximize the number of perceptual “hits” at the expense of an increase of “false alarms.”

Perceptual systems are viewed as inherently self-optimizing and will adjust sensitivity/response bias thresholds within the context of the environment and limitations imposed on the system (Lynn & Barrett, 2014). It has recently been proposed that neural systems will reorganize themselves when limits are imposed upon them, in order to maximize their evolutionary “fitness” (Yamaguti & Tsuda, 2015). Thus, the observed differences in hallucinating participants in the current study could be conceptualized as a compensatory response in the face of Lewy body pathology.

Introducing a prime stimulus had only a small and unreliable (in the case of VH–/VH+ participants) effect on sensitivity. This suggests that environmental cues do not significantly improve sensitivity to ambiguous information. However, the introduction of a prime item did eliminate significant differences in response bias between the groups. In addition, examination of pareidolia rate indicated that the presence of a prime reduced the number of illusory perceptions experienced by hallucinating participants, although their absolute number remained higher than the other groups. These converging findings provide some evidence for the ameliorative effects of environmental cues on frequency of illusory perceptions, although given the limited nature of these findings, this conclusion can only be tentative.

Priming visual environments to reduce illusory perceptions might have practical implications for the care of LBD patients; reducing visual noise and increasing environmental cues may lead to a reduction in hallucination frequency. Guidance on the design of “dementia-friendly” environments is already in existence (Fuggle, 2013) and further research into this area may offer new insights to develop these strategies further in order to discover what kind of visual cues are most effective. It should be noted, however, that in the present study, even after priming, hallucinating participants still had a lower sensitivity score than their non-hallucinating counterparts, as well as a higher number of pareidolia. While environmental cues may therefore play a role in reducing the frequency of illusory perceptions, it is unlikely that they will go as far as to eliminate them.

There are some limitations in the study that indicate a need for caution. First, the sample size was modest (although comparable to similar studies), meaning that the study might have lacked sufficient power to detect some significant results. In addition, while the groups were matched on age, education level, and number of medications taken (in the case of the patient groups), they differed on cognitive ability, visual acuity, disease duration, and diagnosis (there was a higher proportion of patients with Parkinson’s dementia and dementia with Lewy bodies in the hallucinating group). There were also significantly more men in the hallucinating group. The influence of these variables cannot be ruled out.

The differences in visual acuity between hallucinating and non-hallucinating groups is of note. The significant drop in sensitivity score in hallucinating patients may have been related to the significantly poorer visual acuity in this group, rather than due to the neurocognitive changes that are associated with LBD. However, the visual acuity of “low acuity” non-hallucinating patients was indistinguishable from acuity scores of hallucinating participants, yet sensitivity scores remained significantly lower in the hallucinating group. We would contend that this evidence goes some way to ruling out the confounding variable of visual acuity.

In conclusion, pareidolia offer a useful experimental tool to investigate the mechanisms of visual hallucinations. The current study incorporated this class of stimulus with a priming paradigm in order to assess top-down and bottom-up factors in visual hallucinations in LBD. Extending previous research, the findings suggest that only people who hallucinate are poor at discerning visual signals from visual noise. In addition, this deficit in perception appears to be compensated for by a more liberal detection threshold, enhancing the likelihood of perception at the cost of simultaneously enhancing the rate of hallucinatory perception. Environmental factors such as visual cues may therefore potentially modulate the chance of hallucinations.

## Additional file

Additional file 1: Pareidolia Dataset. (XLSX 31 kb)

## Abbreviations

HC: Healthy control participants
LBD: Lewy body disease
MoCA: Montreal Cognitive Assessment
UPDRS: Unified Parkinson’s Disease Rating Scale
VH–: Participants with Lewy body disorder without visual hallucinations
VH+: Participants with Lewy body disorder with visual hallucinations
